# Pharmacological Inhibition of Dynamin II Reduces Constitutive Protein Secretion from Primary Human Macrophages

**DOI:** 10.1371/journal.pone.0111186

**Published:** 2014-10-27

**Authors:** Maaike Kockx, Denuja Karunakaran, Mathew Traini, Jing Xue, Kuan Yen Huang, Diana Nawara, Katharina Gaus, Wendy Jessup, Phillip J. Robinson, Leonard Kritharides

**Affiliations:** 1 Atherosclerosis Laboratory, ANZAC Research Institute, University of Sydney, Sydney, Australia; 2 Centre for Vascular Research, University of New South Wales, Sydney, Australia; 3 University of Ottawa Heart Institute, Ottawa, Canada; 4 Children’s Medical Research Institute, University of Sydney, Sydney, Australia; 5 Department of Cardiology and ANZAC Research Institute, Concord Hospital, University of Sydney, Sydney, Australia; Institut Curie, France

## Abstract

Dynamins are fission proteins that mediate endocytic and exocytic membrane events and are pharmacological therapeutic targets. These studies investigate whether dynamin II regulates constitutive protein secretion and show for the first time that pharmacological inhibition of dynamin decreases secretion of apolipoprotein E (apoE) and several other proteins constitutively secreted from primary human macrophages. Inhibitors that target recruitment of dynamin to membranes (MiTMABs) or directly target the GTPase domain (Dyngo or Dynole series), dose- and time- dependently reduced the secretion of apoE. SiRNA oligo’s targeting all isoforms of dynamin II confirmed the involvement of dynamin II in apoE secretion. Inhibition of secretion was not mediated via effects on mRNA or protein synthesis. 2D-gel electrophoresis showed that inhibition occurred after apoE was processed and glycosylated in the Golgi and live cell imaging showed that inhibited secretion was associated with reduced post-Golgi movement of apoE-GFP-containing vesicles. The effect was not restricted to macrophages, and was not mediated by the effects of the inhibitors on microtubules. Inhibition of dynamin also altered the constitutive secretion of other proteins, decreasing the secretion of fibronectin, matrix metalloproteinase 9, Chitinase-3-like protein 1 and lysozyme but unexpectedly increasing the secretion of the inflammatory mediator cyclophilin A. We conclude that pharmacological inhibitors of dynamin II modulate the constitutive secretion of macrophage apoE as a class effect, and that their capacity to modulate protein secretion may affect a range of biological processes.

## Introduction

Dynamin II belongs to a family of large GTP-binding proteins involved in membrane fission. There are three mammalian classical dynamins: Dynamin I, which is primarily expressed in brain; dynamin II which is ubiquitously expressed; and dynamin III which is expressed predominantly in neurons and testes [1], [2]. Dynamin proteins contain a number of conserved domains: a GTPase domain for GTP hydrolysis; a pleckstrin homology (PH) domain mediating lipid binding; a GTPase effector domain (GED); a middle domain which together with the GED domain controls self-assembly; and a proline-rich domain (PRD) for interacting with SH3 domain-containing proteins [3].

Due to their role in membrane dynamics, dynamins play an important role in vesicle generation during endocytosis, in mitosis and exit from the Golgi [3]–[5]. Although the role of dynamin II in endocytosis is clearly established, its precise role in constitutive protein secretion, especially in the delivery of proteins from the Golgi to the plasma membrane, is less clear. Kasai et al found no effect of GTPase-deficient dynamin II mutant K44A (dynIIK44A) on exocytic transport of Cathepsin D and thermoreversible Vesicular Stomatitis Viral Glycoprotein (VSVG) [6]. Similarly, Altschuler et al [7] showed normal transport of the transferrin receptor and polymeric Ig receptor in cells transfected with dynIIK44A. In contrast, Weller et al and Liu et al found that transport of VSVG from the Golgi to the plasma membrane was blocked by dynIIK44A and by dynamin II mutants that cannot be phosphorylated [5], [8]. The apparent discrepancy may be related to variations in the cell types studied. For example, the transport of VSVG was found to be mediated by dynamin in some cells, and by another fission protein, carboxy-terminal binding protein 3/brefeldin A-ribosylated substrate (CtBP3/BARS) in other cell types [9].

The search for inhibitors of endocytosis and new anti-mitotics for cancer therapy led to the discovery of several classes of cell permeable small molecules that effectively inhibit dynamin activity. The first reported dynamin inhibitors were long chain ammonium salts [10] with myristyl trimethyl ammonium bromide (MiTMAB) and octadecyltrimethyl ammonium bromide (OctMAB) the most potent among this class. MiTMABs inhibit dynamin activity by interfering with binding of the PH-domain to phospholipids thereby blocking dynamin recruitment to membranes [11].

A second class of dynamin inhibitors are the “dynasore” molecules. Dynasore was identified in a screen of 16,000 small molecules as an inhibitor of dynamin I, dynamin II and dynamin related protein 1 that interfered with the GTPase activity of dynamins in a non-competitive manner [12]. Subsequently, more potent dynasore reagents termed the Dyngo analogues were developed [13]. A third group of compounds, the Dynole series were also found to non-competitively inhibit dynamin GTPase activity. Of these, dynole 34-2 (2-cyano-3-(1-(2-(dimethylamino)-ethyl)-1*H*-indol-3-yl)-*N*-octylacrylamide) is the most potent [14].

Although, effective inhibition of endocytosis by these dynamin inhibitors has been shown [11], [14]–[16] their effect on constitutive protein secretion, a process requiring intact membrane dynamics, is unknown. ApoE is a constitutively secreted mammalian glycoprotein which plays an important role in the pathogenesis of atherosclerosis and Alzheimers’ Disease [17]. Secretion of apoE occurs through the classical secretory pathway, involving transport from the ER to Golgi followed by budding from the trans Golgi Network and transport to the plasma membrane [18]. ApoE is understood to protect against amyloid fibril formation and self aggregation [19], demonstrates anti-inflammatory activity [20], and in particular apoE released from macrophages is lipid-lowering and anti-atherosclerotic [21]. Modulation of apoE secretion by modifying protein kinase C activity concurrently affects the secretion of other proteins such as fibronectin, chitinase-3-Like protein 1 (CHI3L1), Matrix Metalloproteinase 9 (MMP9), and lysozyme [22], implying that the mediators of apoE secretion may be of broad relevance to a range of constitutively secreted proteins.

We here investigate the role of dynamin in regulating constitutive secretion of apoE and other proteins, comparing the effects of structurally distinct classes of pharmacological dynamin inhibitors and investigate likely mechanisms of action.

## Materials and Methods

### Materials

Dynasore was obtained from Sigma. Low density lipoprotein (LDL) and acetylated LDL (AcLDL) were prepared as previously described [23]. Stealth RNA oligo’s targeting dynamin II (stealth_1318 and stealth_514) were obtained from Invitrogen. Tetradecylamine (TDA); myristyl trimethyl ammonium bromide (MiTMAB); octadecyltrimethyl ammonium bromide (OctMAB), Dyngo-4a, Dyngo-7a and Dynole-34-2 were sourced as previously reported [13], [14]. Dyngo-4a, Dyngo-7a and Dynole-34-2 are trademarks of Newcastle Innovation and Ltd. and Children’s Medical Research Institute and are available from Abcam Biochemicals Ltd (Cambridge, UK).

### Cells and drug exposure

Primary human monocyte-derived macrophages (HMDM) were generated from buffy coats of unknown gender, obtained from the Australian Red Cross Blood Service, as described [24]. Cells were cultured in RPMI 1640 containing 10% (v/v) heat inactivated human serum, penicillin G and streptomycin (50 units/ml and 50 µg/mL, respectively) and 50 ng/mL m-CSF. Cells were cholesterol enriched by incubation with 50 µg/mL acetylated LDL for 48 h to obtain foam cell macrophages before incubation with dynamin inhibitors. Chinese Hamster Ovary (CHO)-K1 cells (ATCC) stably expressing and secreting human apoE (CHO-apoE) under a CMV promoter have been previously described [24]. Human Hepatoma (HepG2) cells (ATCC) were maintained in DMEM containing 10% (v/v) fetal bovine serum, penicillin G and streptomycin (50 units/ml and 50 µg/mL, respectively).

Dynamin I, II and III triple knockout fibroblast cultures were generated and maintained as previously described in Ferguson et al. [25] and Raimondi et al [26]. Cells were incubated with 3 µM 4-hydroxytamoxifen (Sigma) for 2 days, then maintained in the presence of 300 nM 4-hydroxytamoxifen, resulting in dynamin depletion at 5–6 days from the start of the treatment period. TKO cells were used for experiments between 7 and 9 days. Control cells were the triple conditional KO cells without 4-hydroxytamoxifen treatment.

A cell-line stably expressing thermo-reversible vesicular stomatitis virus glycoprotein (VSVGt) linked to GFP was previously described [27]. In short, SRD-13A cells, a CHO cell-line deficient in SCAP, were co-transfected with pGFP-VSVG and a plasmid encoding SCAP. Cells expressing these plasmids were selected by supplying only lipoprotein-deficient medium.

Dynamin inhibitors were dissolved in DMSO and used within previously reported concentration ranges and at which preliminary experiments had demonstrated were not toxic (see below) after 3 h exposure, with the exception of mouse fibroblasts which only tolerated Dynole-34-2 at 10 µM concentration. Cells were incubated with the indicated concentration of dynamin inhibitors or corresponding concentration of DMSO vehicle (0.1% v/v) in media containing 0.1% (w/v) BSA.

Cell viability was routinely monitored by light microscopy and was quantified by assay of lactate dehydrogenase (LDH) release [23].

### Determination of protein and mRNA levels

ApoE secreted into the medium was measured by ELISA and confirmed by Western Blot analysis as described previously [23]. Secretion and cellular levels of apoE and other macrophage proteins were determined by Western blotting with the following antibodies: apoE (Meridian lifesciences), fibronectin (BD biosciences), matrix metalloproteinase 9 (MMP9; abcam) clathrin heavy chain (CHC; BD biosciences), heat shock protein 90 (HSP90; BD biosciences), chitinase-3-like protein 1 (CHI3L1; R & D systems), cyclophilin A (CypA; Abcam) dynamin I (Abcam) and dynamin II (Abcam).

Total RNA was isolated, and apoE mRNA levels were analyzed by quantitative real time-PCR as described previously [23].

### Two-Dimensional Gel electrophoresis (2-DE)

To detect individual apoE glycoforms, apoE was subjected to 2-DE as described previously [28].

### [^35^S]-Methionine/Cysteine protein labeling

Metabolic labeling was carried out as previously reported [23]. In short, cells were incubated with methionine/cysteine free DMEM (Invitrogen) containing 250 µCi/mL [^35^S]methionine/cysteine (Perkin Elmer) for 3 h and received a pre-incubation with 30 µM MiTMAB during the last 30 min. Cells were then washed twice and incubated with RPMI containing 0.1% BSA and 2 mM L-methionine and L-cysteine for 2 h. [^35^S]-labeled apoE in cell lysates and medium at indicated time points was immunoprecipitated using a goat antibody to human apoE (MilliPore) and protein A-Sepharose (Amersham) and was separated by SDS-PAGE. The 34 kDa band was quantified by phosphorimaging (Photostimulated Luminescence, Fujix BAS-1000). Total protein secreted was determined by TCA-precipitation and scintillation counting.

### Live cell microscopy

Live cell imaging was performed as described previously [22]. In short, cultured HMDM (2×10^5^ cells/mL) were transiently transfected with 3–5 µg of apoE-GFP cDNA using the Amaxa transfection system according to the manufacturer’s instructions and incubated overnight. Live cell imaging of apoE-GFP positive HMDM for 3–5 mins was performed using the Zeiss LSM 780 confocal microscopy equipped with x63 water-immersion lens, a heated stage, a temperature and CO_2_ regulated incubator. Untreated GFP-positive cells were imaged for 3–5 mins prior to incubating the cells with 30 µM MiTMAB or Dynole 34-2 for 15–30 min prior to imaging the same cell. The average speed of 140 representative apoE-GFP containing vesicles in different cells from at least 3 independent buffy coat preparations was quantified using Imaris software (v7.2.3; Bitplane AG).

### Microscopy

HMDM were treated with indicated dynamin inhibitors for 2 h, fixed in ice cold methanol, blocked in phosphate buffered saline (PBS) containing 1% goat serum, stained for α-tubulin (sigma) followed by secondary anti-mouse Alexa 488 FAB fragments (Cell signalling) in blocking buffer. Slides were washed, then mounted in a mixture of anti-fading media Mowiol 4-88 (Calbiochem) and glycerol, with the addition of DABCO (1,4-diazobicyclo-[2.2.2]-octane) (Sigma). Images were captured using an Olympus FV1000 confocal laser scanning microscope equipped with a 60x oil immersion objective.

### Endoglycosidase H (Endo H) cleavage

CHO-GFP-VSVGt cells were lysed in water and treated with Endo H using recombinant Endo H (New England Biolabs) according to the manufacturer’s instructions. Briefly, proteins were denatured and incubated overnight at 37°C with 50 units Endo H. After termination of the reaction, cleavage of VSVG was assessed by Western Blotting using rabbit anti-GFP (LifeTechnologies).

### Data analysis

The degradation and secretion of cellular apoE in pulse-chase experiments were simultaneously fitted to a first-order rate with k_1_ and k_2_ describing the rate constants for secretion and degradation, respectively as described previously [23], [24], [29].
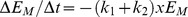
(1)


This equation (Eq. 1) was fitted to the experimental secretion and degradation data using a non-linear least-squares fitting program (Solver, Microsoft Excel). The quality of the fit was evaluated by an error function as described. Cellular apoE was previously shown to exist in stable (Es) and mobile pools (Em).

Data presented are the mean ± SEM of independent experiments using 3 or more different macrophage donors unless otherwise indicated. Comparison of pulse-chase time course experimental data was performed using two-way repeated measures analysis of variance with exposure to control or MiTMAB as the between group effect. Comparisons of two groups were performed by Mann Whitney U test as appropriate. A significant difference between control and multiple treatment groups was assessed by analysis of variance using Dunnett’s post hoc test for multiple comparisons. Differences were considered significant at *P*<0.05.

## Results

### Inhibition of dynamin inhibits secretion of apoE from primary human macrophages

**Figure 1:**
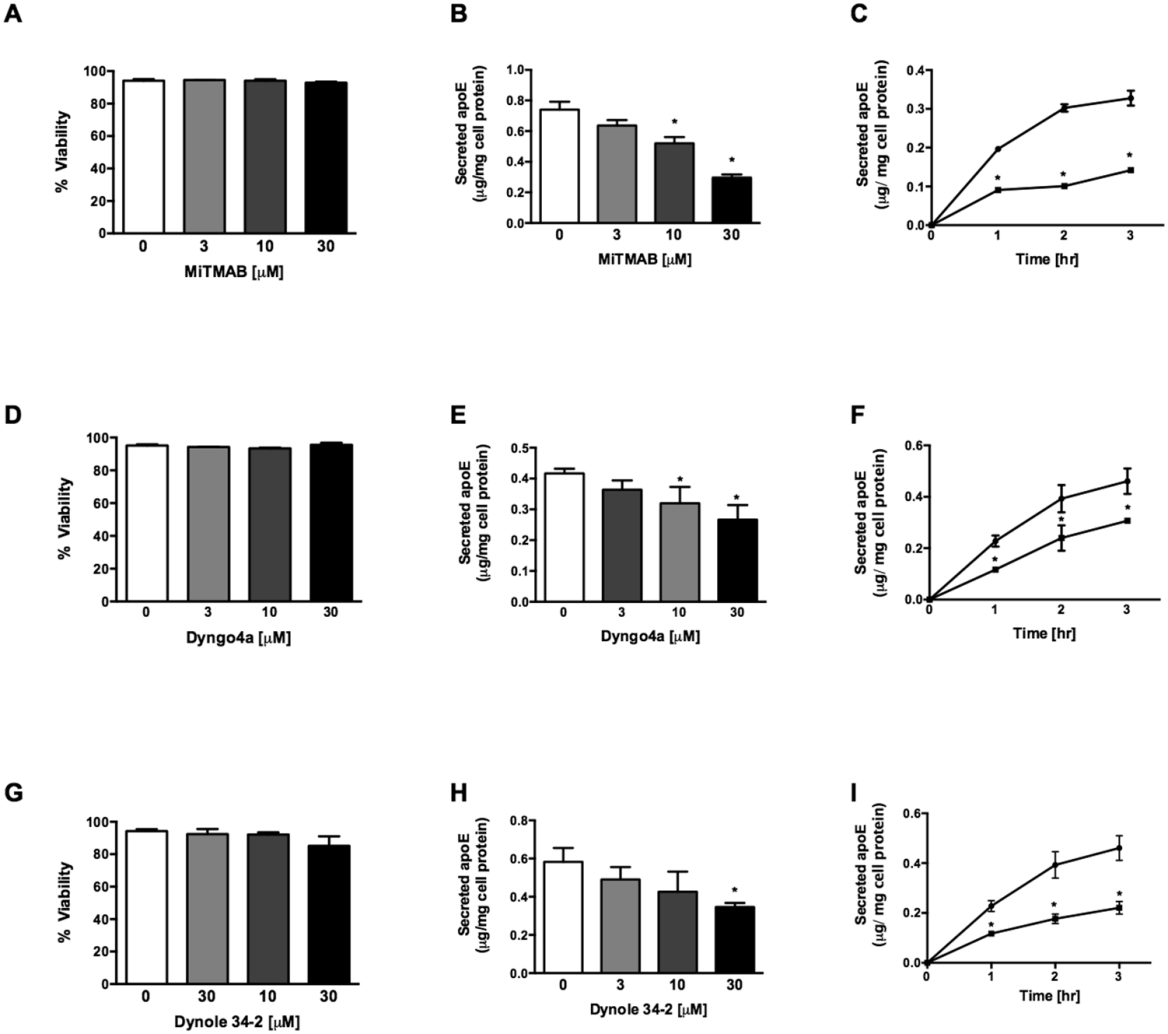
Dynamin inhibition decreases apoE secretion from primary human macrophages. HMDM were incubated with indicated concentrations for 3 h (A and B) and for indicated time periods at 30 µM (C) with a representative compound for each class of dynamin inhibitor. MiTMAB (A–C), Dyngo-4a (E–F) and Dynole-34-2 (G–I). Cell viability and secreted apoE were measured by LDH assay and ELISA, respectively. (•) Ctrl and (▪) Treated. Data shown are mean ± SD from triplicate cultures representative of at least 3 individual donors. *P<0.05 *v* Ctrl.

To test whether dynamin inhibition affected the secretion of apoE from primary human macrophages, HMDM were exposed to various classes of cell permeable dynamin inhibitors at concentrations found in preliminary experiments to be compatible with normal cell viability. Long chain amines and ammonium salts, which inhibit dynamin recruitment to membranes, effectively inhibited the secretion of apoE from HMDM (Table 1). Inhibition of apoE secretion was both dose and time-dependent, under conditions that did not affect HMDM viability (Fig. 1A–C data for MiTMAB). Non-competitive inhibitors of dynamin GTPase activity, the dynasore analogues and Dynole-34-2, also inhibited the secretion of apoE from HMDM (Table 1). Dyngo-4a, belongs to the group of dynasore-related inhibitors and Dynole-34-2 both dose- and time-dependently decreased apoE secretion without affecting HMDM viability (Fig. 1D–F and 1G–I). Of all inhibitors tested, the long chain amines and ammonium salts were the most potent, with MiTMAB inhibiting apoE secretion by 45–60% after 2 h incubation.

**Table 1:** Inhibition of apoE secretion by dynamin inhibitors.

Inhibitor class	% inhibition	*P<*
*Long chain amines*		
TDA (30 µM)	51±13	0.05
MiTMAB (30 µM)	60±2	0.0001
OcTMAB (30 µM)	46±7	0.0001
*Dynasore and analogues*		
Dynasore (80 µM)	39±7	0.001
Dyngo-7a (30 µM)	34±4	0.0001
Dyngo-4a (30 µM)	36±6	0.0001
*Indoles*		
Dynole-34-2 (3 0 µM)	44±9	0.01

HMDM were exposed to inhibitors for 2 hours. Data are mean ± SEM of three independent cell donors.

TDA, tetradecylamine; MiTMAB, myristyl trimethyl ammonium bromide; OcTMAB, octadecyltrimethyl ammonium bromide.

**Figure 2:**
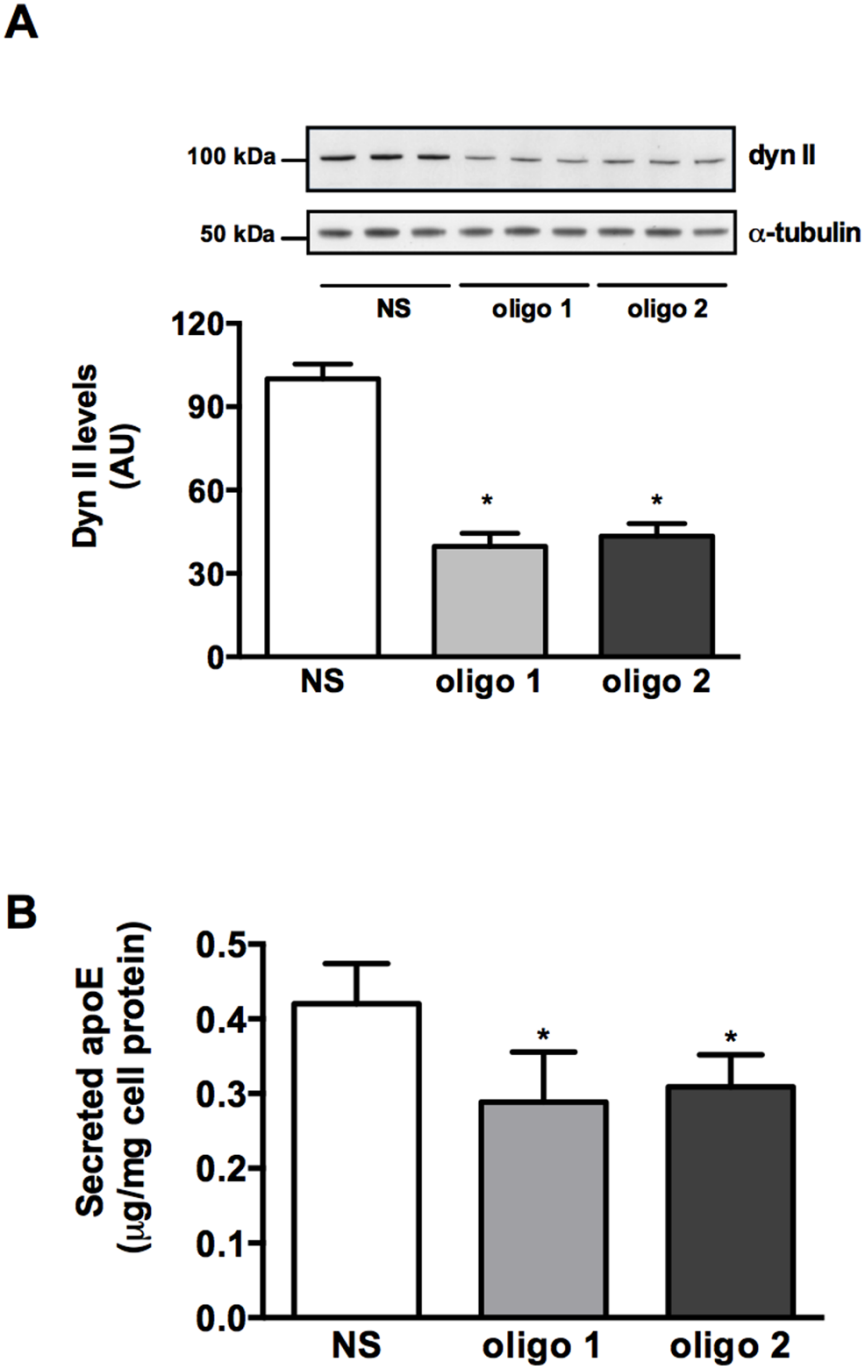
Silencing of dynamin II decreases apoE secretion. HMDM were incubated with 150 nM non-silencing or dyn II siRNA oligo’s for 48 h, followed by a further incubation for 72 h in RPMI containing 10% LPDS and 50 µg/mL AcLDL. After loading apoE secretion was determined over a 2 h period in 0.1% BSA in RPMI. Cellular dynamin II levels (A) and secreted apoE (B) were determined by Western blotting and ELISA, respectively. Data are mean ± SD from triplicate cultures representative of at least 2 individual donors. *P<0.05 *v* Ctrl.

To confirm that these effects may be attributable to inhibition of dynamin II, and not off target effects, cellular dynamin II was silenced using siRNA. Several protocols were tested and a maximum knockdown of dynamin II levels of 60% was achieved 72 h after incubation with both siRNA oligos tested (Fig. 2A). Under these conditions apoE secretion was inhibited by 25±7% (p = 0.01; Fig. 2B). Taken together with the uniform effects of a structurally diverse range of dynamin inhibitors these data indicate that dynamin II is involved in the secretion of apoE from primary human macrophages.

### Dynamin inhibitors affect apoE secretion post-translationally

**Figure 3:**
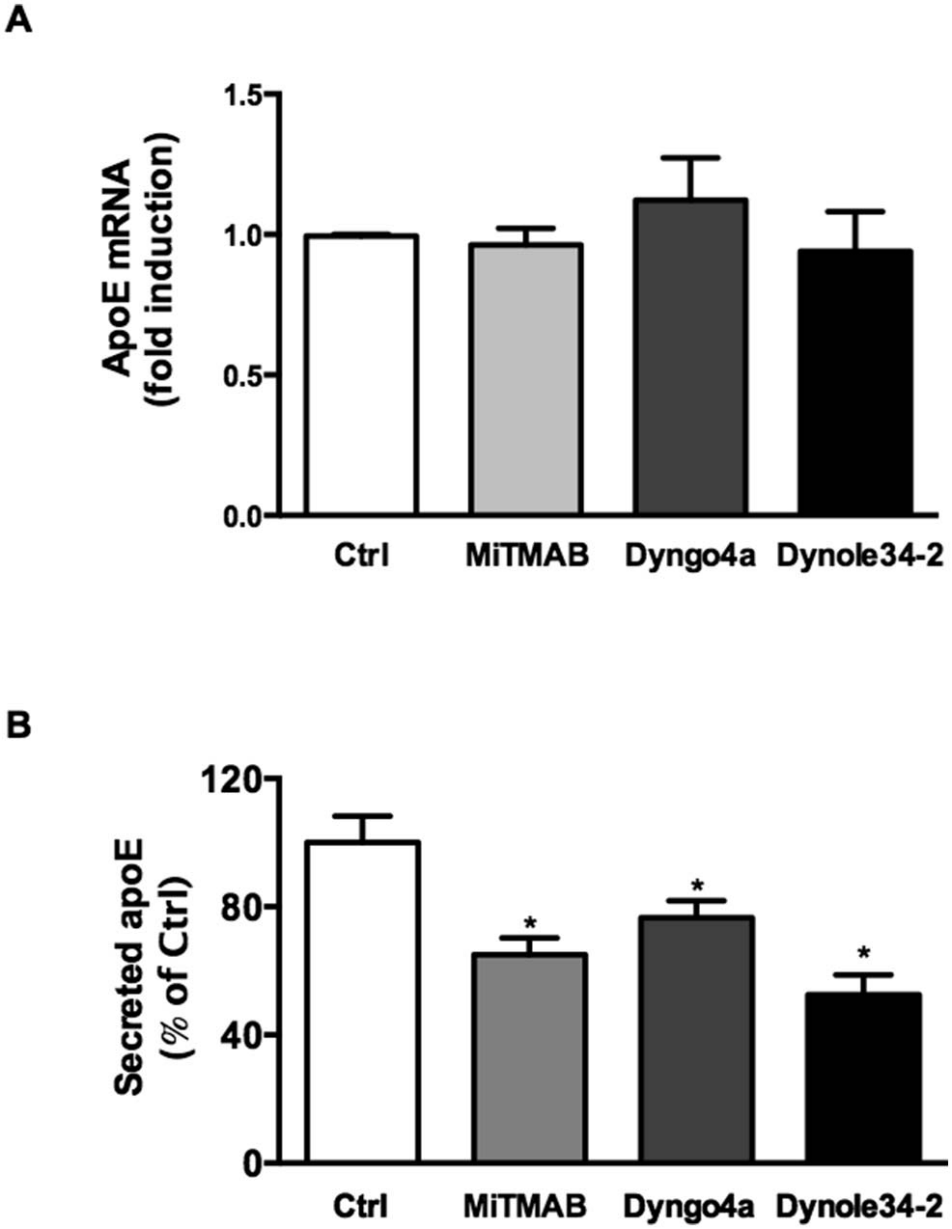
Dynamin inhibition affects apoE post translationally. HMDM were incubated with 30 µM MiTMAB, Dyngo-4a or Dynole-34-2 for 2 h and cellular apoE mRNA levels (A) were determined by real time PCR. CHO-apoE cells (B) were incubated with 30 µM MiTMAB, Dyngo-4a, or Dynole-34-2 for 2 h and secreted apoE levels were determined by ELISA. Data shown in (A) are mean ± SD from triplicate cultures representative of at least 2 individual donors for HMDM while panel B are mean ± SEM from 3 experiments.

To determine whether decreased apoE secretion was mediated by effects on apoE transcription, the effects of MiTMAB, Dyngo-4a and Dynole-34-2 on apoE mRNA levels were determined. None of the inhibitors affected apoE mRNA levels (Fig. 3A), suggesting decreased apoE secretion is not mediated via effects on apoE production. In addition, we tested dynamin inhibition in Chinese Hamster Ovary cells stably expressing human apoE under a CMV promoter (CHO-apoE). Previous studies have shown that apoE secretion from these cells is under similar post-transcriptional regulation as apoE secreted from macrophages [24]. All inhibitors tested decreased the secretion of apoE from CHO-apoE cells (Fig. 3B), indicating that dynamin regulates secretion of apoE post translationally.

### Dynamin inhibition directly affects apoE secretion and degradation

**Figure 4:**
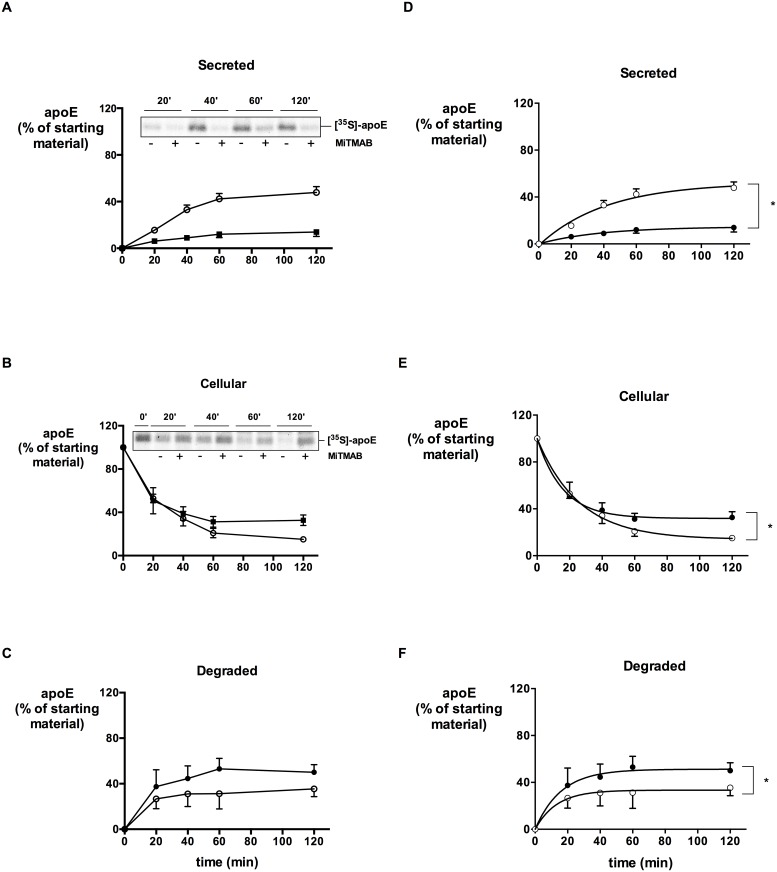
Dynamin inhibition affects secretion and degradation of pre-labeled [^35^S]-apoE. HMDM were incubated in methionine/cysteine free DMEM with 250 µCi/mL [^35^S]methionine/cysteine for 3 h and received a preincubation with or without 30 µM MiTMAB during the last 30 min. Cells were then washed and chased in medium containing unlabelled methionine/cysteine, without or with 30 µM MiTMAB. At the indicated times [^35^S]-labelled apoE was immunoprecipitated from media and cell lysates, separated by SDS-PAGE and quantified by phosphorimaging. Secreted [^35^S]-apoE (A); cell-associated [^35^S]-apoE (B); net degradation of [^35^S]-apoE (C), calculated by subtracting residual [^35^S]-apoE in cell and media from [^35^S]-apoE at T_0_. Inserts show representative images. Fitted data according to Eq1 are shown (D–F). Symbols Ctrl (^○^) and MiTMAB (•) represent experimental data. *P<0.05 between treatments (Repeated Measures 2-way ANOVA).

To confirm that dynamin directly inhibited secretion of apoE, independent of any effects on the synthesis of apoE we used a pulse-chase metabolic labeling protocol, and MiTMAB as the prototype dynamin inhibitor. Using this protocol, we have previously performed kinetic modeling on the secretion and degradation of [^35^S]-labelled apoE and showed that cellular apoE exists in a relatively small stable (Es) and a larger mobile (Em) pool, and that the secretion and degradation can be described with a first order rate Eq1 with rate constants k_1_ and k_2_ for secretion and degradation, respectively [23]. In the presence of MiTMAB the secretion of [^35^S]-labelled apoE was markedly decreased (Fig. 4A, p<0.0001), which was reflected by a decreased rate constant k_1_ after fitting the experimental data to the first order rate (Table 2). Cellular [^35^S]-apoE levels initially declined at the same rate as Ctrl cells, but declined more slowly at later time points (Fig. 4B, p = 0.01). Interestingly, cellular [^35^S]-apoE levels were higher after MiTMAB treatment than controls, indicating that a proportion of apoE which is not secreted as a result of dynamin inhibition accumulates intracellularly (Fig. 4B). Net degradation of apoE (determined by subtracting [^35^S]-apoE present in medium and cells at each time point from the initial amount of [^35^S]-apoE in cells at t = 0) was increased by MiTMAB (Fig. 4C, p = 0.0008 and increase in k_2_ in Table 2) indicating that a significant proportion of apoE which was not secreted because of MiTMAB had been degraded. The accumulation of apoE in the cells was associated with an increase in the size of the stable pool (Es Table 2). The curves derived from the modeling parameters showed excellent fit to the raw data (Fig. 4D–F and ERF <0.05 for both control and MiTMAB). Taken together these data indicate that MiTMAB inhibits secretion of a prelabeled pool of apoE, with most of the intracellularly retained apoE being degraded and presumably redirected to a proteolytic compartment.

**Table 2:** Summary of modeling parameters for apoE secretion and degradation.

	Ctrl	MiTMAB	*P*
k_1_ (min^−1^)	0.019±0.002	0.008±0.004	0.01
k_2_ (min^−1^)	0.016±0.012	0.036±0.017	0.03
Es (%)	14.2±4.4	32.8±10.9	0.04
ERF	0.013	0.0016	

HMDM were incubated in methionine/cysteine free DMEM with 250 µCi/mL [^35^S]methionine/cysteine for 3 h and received a preincubation of 30 µM MiTMAB during the last hour. Cells were then washed and chased in medium containing unlabelled methionine/cysteine, without or with 30 µM MiTMAB. At the indicated times [^35^S]-labelled apoE was immunoprecipitated from media and cell lysates, separated by SDS-PAGE and quantified by phosphorimaging. Data are mean ± SD from 3 independent experiments performed in duplicate. k_1_ and k_2_ represent the secretion and degradation rate constants, respectively. Es represents the percent of cellular apoE in the stable pool, ERF represents the error function of modelling parameters. All data are expressed as percent of total [^35^S]-apoE at *T*_0._ p values were determined by Student’s *t*-test.

### Dynamin inhibition affects apoE transport after processing in the Golgi compartment

**Figure 5:**
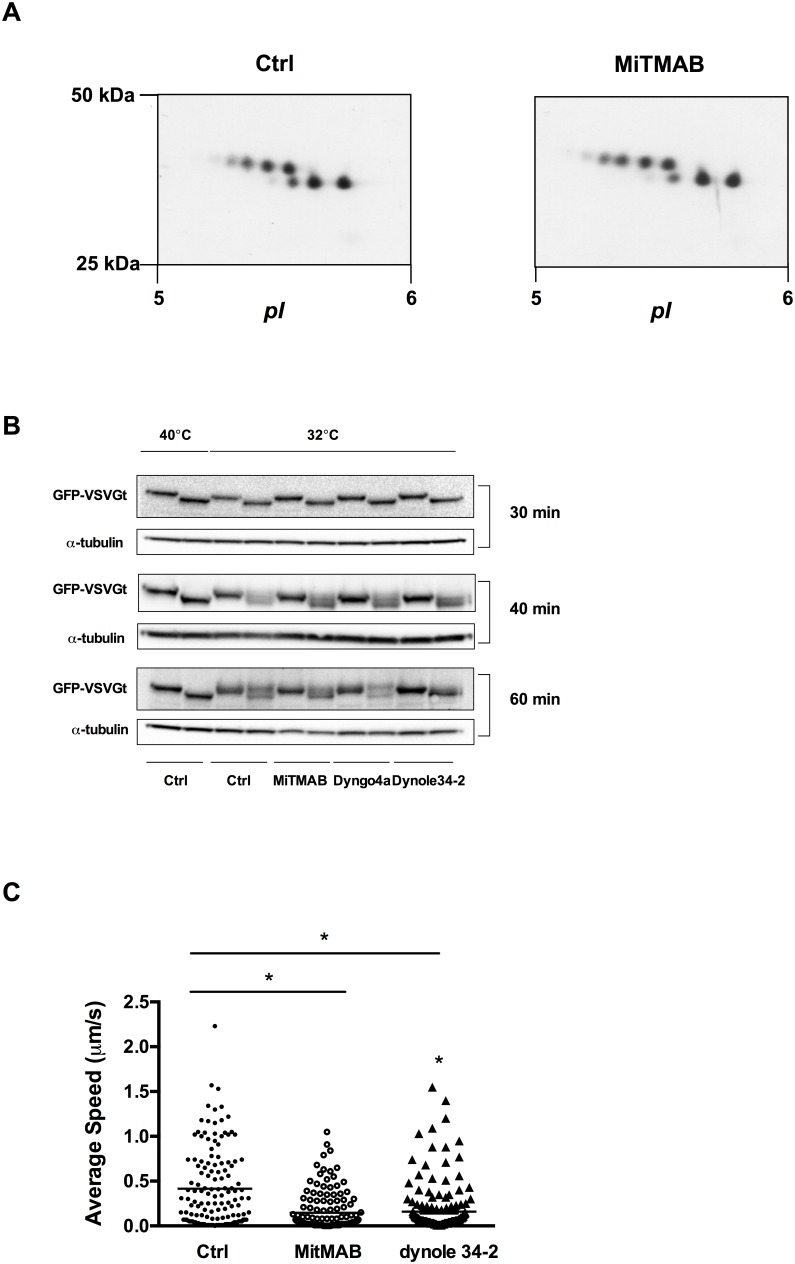
Dynamin inhibition affects post-Golgi secretion and movement of apoE-containing vesicles. (A) HMDM were treated with ±30 µM MiTMAB for 2 h and cellular ApoE glycoform distribution was determined by 2D-GE and Western blotting. (B) CHO-GFP-VSVGt cells were incubated overnight at 40°C to accumulate VSVG in the ER. Cells were then exposed to 30 µM MiTMAB, Dyngo-4a and Dynole-34-2 for indicated time points at 32°C. Cell lysates were harvest and cleavage of VSVGt by Endo H assessed as described in Material and Methods. GFP-VSVGt (≈120 kDa) and α-tubulin (52 kDa) as a loading control are shown for each time point. (C) HMDM were transiently transfected with apoE-GFP and cultured for 24 h prior to performing live cell microscopy. Individual cells expressing apoE-GFP were identified using a Zeiss LSM 780 microscope equipped with a heated stage and CO_2_ chamber, and GFP-positive vesicles were tracked for 3–5 min. HMDM were then treated with 30 µM MiTMAB or Dynole-34-2 for 15–30 min prior to imaging. Representative movies are shown in the Supplement. Average speed was quantified using Imaris software and 20 representative spots were randomly selected from 7 cells from at least 3 individual donors. *P<0.0001 *v* Ctrl.

ApoE is extensively glycosylated and sialylated during its transport from the ER to the Golgi [28] and its glycosylation/sialylation pattern gives an indication of whether transport of apoE is inhibited before or after exit from processing in the Golgi [27], [30]. Analysis of apoE glycoforms by 2D-gel electrophoresis showed identical apoE glycoform patterns between control and MiTMAB treated cells (Fig. 5A) or after Dyngo-4a and Dynole-34-2 exposure (data not shown), indicating that dynamin inhibition affects apoE secretion after it is fully glycosylated and sialylated in the Golgi.

Thermo-reversible transmembrane VSVG protein is widely used to study intracellular protein traffic. At 40°C VSVGt reversibly misfolds, resulting in ER accumulation. Traffic can resume at 32°C with VSVG moving from the ER to the Golgi complex. Under normal conditions, processing of *N*-glycans in the Golgi renders VSVGt resistant to Endo H cleavage [31]. To further investigate whether intracellular protein traffic is affected by dynamin inhibitors, cells were incubated overnight at 40°C, then treated with 30 µM MiTMAB, Dyngo-4a or Dynole-34-2. At indicated time points, cells were harvested and cleavage of VSVG by Endo H assed. Endo H treatment of cells after overnight incubation at 40°C showed that all VSVGt present was accessible to Endo H (Fig. 5B), consistent with ER localization of VSVGt. No Endo H resistant portion was observed after 20 (not shown) or 30 min incubation at 32°C (Fig. 5B). At 40 min a proportion of VSVGt was resistant to cleavage by Endo H confirming traffic from ER through to the Golgi. Endo H resistance was observed with all 3 classes of dynamin inhibitor used, suggesting dynamin inhibition did not interfere with transport of VSVGt from ER through the Golgi.

We next employed live cell microscopy to determine whether dynamin inhibition interferes with the transport of apoE-containing vesicles. As previously described, apoE-GFP demonstrates a typical peri-nuclear Golgi distribution. ApoE was present in vesicular structures throughout the cell that moved from the Golgi to the plasma membrane and with multi-directional trajectories (Movie S1 [22], [29]). After exposure to MiTMAB, vesicle movement slowed significantly with some vesicles becoming almost stationary (Movie S2). Quantification of the average vesicle speed demonstrated that MiTMAB decreased average vesicle speed significantly from 0.5±0.05 to 0.15±0.02 µm/s Fig. 5C (p<0.0001). Vesicle movement was significantly decreased by a similar extent during exposure to Dynole-34.2 to 0.16±0.02 µm/s (Movie S3, S4 and Fig. 5C). These results indicate that inhibition of dynamin primarily affects apoE transport post-Golgi and decreases the speed of apoE-containing vesicles.

### The inhibition of apoE secretion does not correlate with changes to microtubule morphology or tubulin acetylation

**Figure 6:**
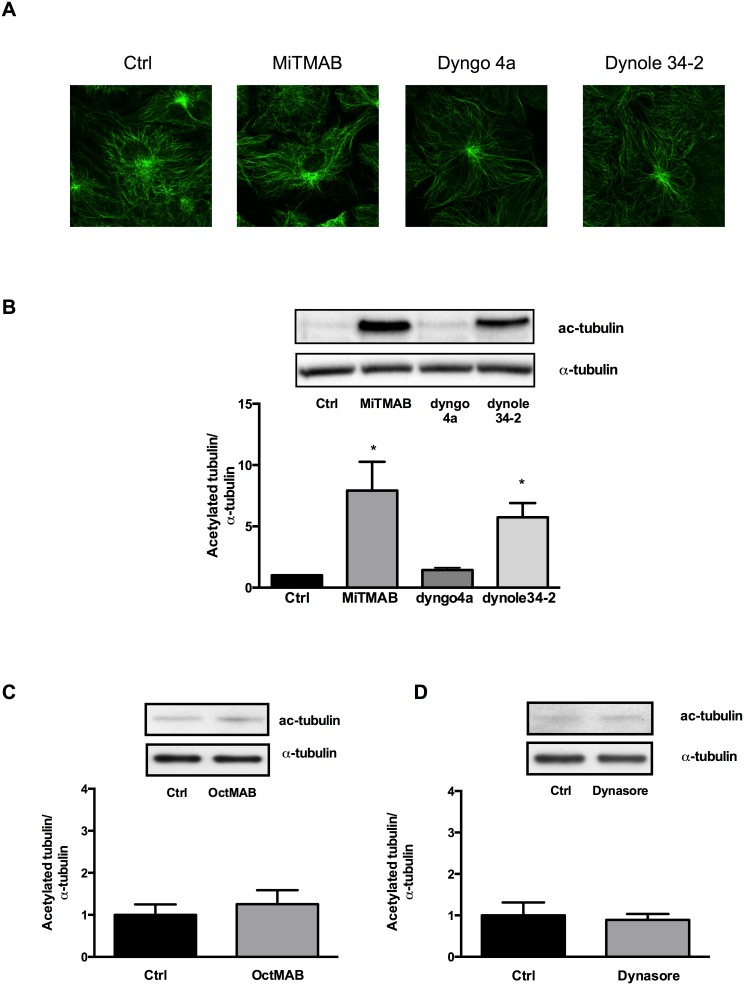
Effect of dynamin inhibitors on microtubule morphology and tubulin acetylation. HMDM were treated without or with 30 µM MiTMAB, Dyngo-4a, Dynole-34-2 (A), OctMAB (C) and 80 µM Dynasore (D) for 2 h. Effects on α-tubulin and acetylated tubulin (Ac-tubulin) were determined by confocal microscopy (A) and Western blotting (B, C and D), respectively. Representative confocal images are shown. Panel B is mean ± SD from at least two individual donors. A representative Western blot is shown. In the above experiments apoE secretion was inhibited by 64, 37, 55, 46, and 34% by MitMAB, Dyngo-4a, Dynole-34-2, OctMAB and Dynasore, respectively *P<0.05 *v* Ctrl.

Dynamin was originally identified as a microtubule-binding GTPase [32]. Various studies indicate that dynamin II controls microtubule dynamics, and siRNA knockdown and mutations in dynamin II increase microtubule stability and cause accumulation of acetylated tubulin, which is a useful marker of enhanced microtubule stability [33]. As we previously showed that the secretion of apoE involves the microtubule network [29], we investigated whether dynamin inhibition altered microtubule morphology or increased levels of acetylated tubulin in human macrophages. Confocal microscopy and Western blotting showed that dynamin inhibitors did not affect either total microtubule morphology or levels of α-tubulin (Fig. 6A). Although we observed a marked increase in acetylated tubulin after treatment with MiTMAB and Dynole-34-2, acetylated tubulin levels were unaffected by incubation with OcTMAB, Dyngo-4a or dynasore (Fig. 6B, C and D). These data argue against a consistent causal role for microtubule stabilisation in mediating the inhibition of apoE secretion achieved by dynamin inhibition.

### Dynamin inhibition decreases secretion of apoE from liver cells

**Figure 7:**
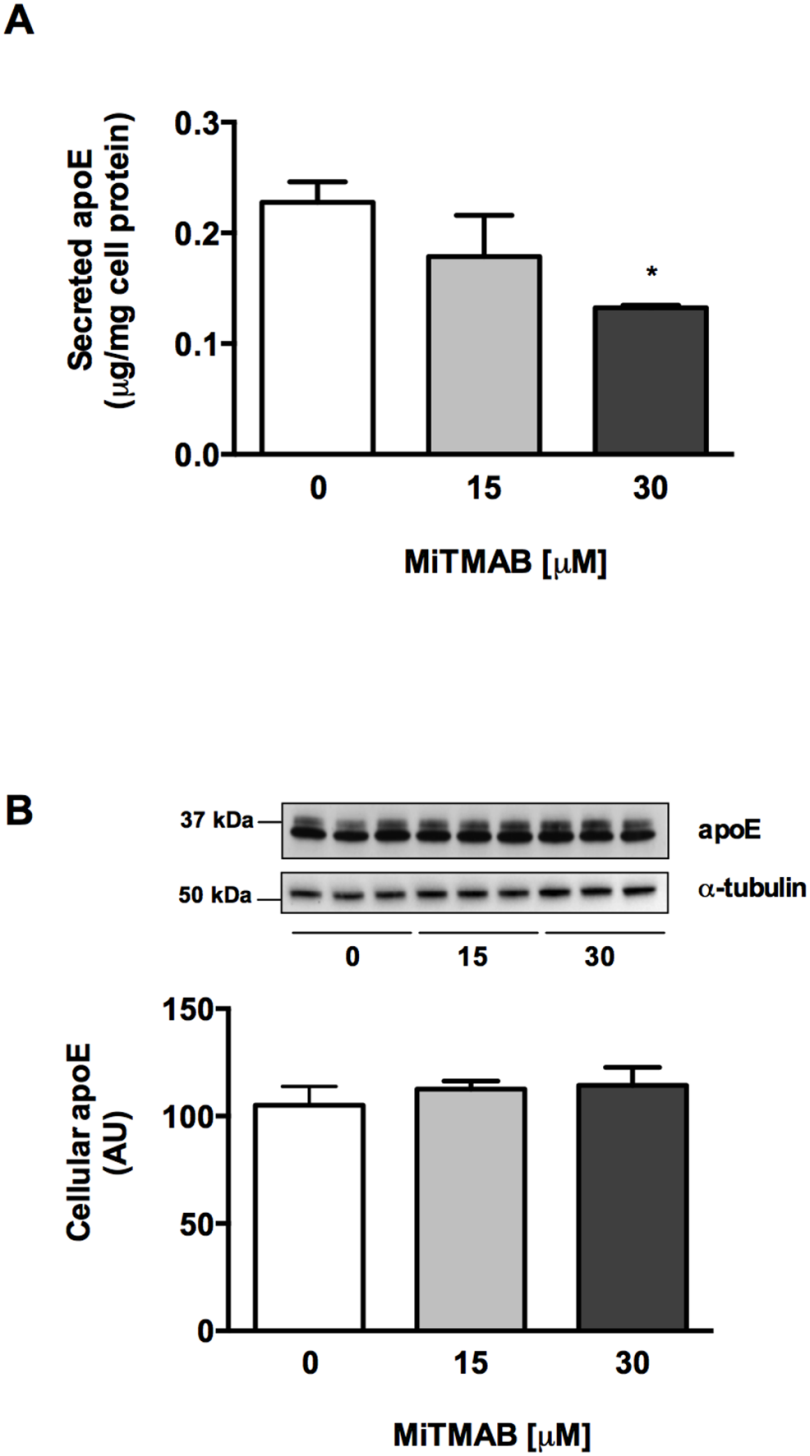
Dynamin inhibition reduces apoE secretion from liver cells. HepG2 cells were treated with indicated concentrations of MiTMAB for 2 h, after which secreted and cellular apoE levels were determined by ELISA and Western blotting. Data shown are mean ± SD from triplicate cultures from one experiment representative of two independent experiments. *P<0.05 *v* Ctrl.

ApoE is also secreted by hepatocytes, and the liver produces most apoE found in circulating blood [17]. To test whether the effect of MiTMAB on apoE secretion was specific to macrophages, we measured the effect of MiTMAB on endogeneous apoE secretion from the human hepatoma cell line HepG2. Similar to HMDM, MiTMAB decreased apoE secretion from HepG2 in a concentration-dependent manner (Fig. 7A) whilst not affecting cellular apoE levels (Fig. 7B), suggesting that dynamin II is involved in apoE secretion from both cell types.

### Dynamin inhibition affects the secretion of proteins other than apoE

**Figure 8:**
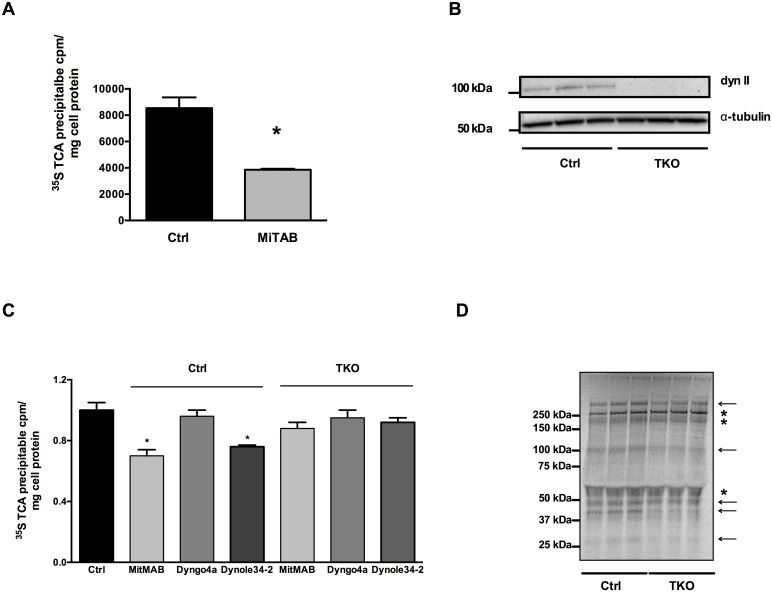
Dynamin inhibition affects secretion of other proteins. HMDM were exposed to 30 µM MiTMAB for up to 2 h. Total protein secreted (A) was determined by TCA-precipitation of [^35^S]-labelled proteins from medium of experiments described in Fig. 4. (B) Ctrl and TKO mouse fibroblasts were generated by treatment without and with 4-hydroxytamoxifen for 7 days, respectively. Cellular Dynamin II levels were determined by Western Blotting and corrected for α-tubulin to account for uneven loading of cell material. (C) Ctrl and TKO mouse fibroblasts were incubated in methionine/cysteine free DMEM with 250 µCi/mL [^35^S]methionine/cysteine for 2 h. Cells were then exposed to 30 µM MiTMAB, 30 µM Dyngo-4a and 10 µM Dynole-34-2 after which total TCA-precipitable counts were determined in medium and cell lysates. Counts are corrected for protein amounts and expressed as a percentage of the initial [^35^S]-precipitable material at T = 0. (D) Phosphorimager image showing [^35^S]- proteins secreted from Ctrl and TKO cells. Lanes were corrected for protein amounts in respective wells and specific activity. Molecular weight markers are annotated. Arrows indicate decreased protein secretion from TKO, asterisks represent proteins with similar protein secretion between Ctrl and TKO cells. Data shown are mean ± SD from triplicate cultures representative of at least 2 experiments. *P<0.05 *v* Ctrl.

We have previously identified that inhibition of Protein Kinase C affected the secretion of apoE as well as several other proteins constitutively secreted from human macrophages [22]. To determine whether the effect of dynamin inhibition was specific for apoE, we measured the effect of MiTMAB on the secretion of other specific constitutively secreted proteins, as well as on total protein secretion using [^35^S]-pulse chase experiments. MiTMAB decreased total TCA-precipitable secreted [^35^S]-material (Fig. 8A) indicating that MiTMAB inhibits overall protein secretion from HMDM. MiTMAB specifically inhibited the secretion of fibronectin, MMP9, CHI3L1 and lysozyme (Fig. S2 and Table 3). However, the secretion of clathrin heavy chain (CHC), Heat Shock Protein 90 (HSP90) were unaffected, while, unexpectedly, the secretion of the inflammatory mediator cyclophilin A (CypA) increased after incubation with MiTMAB. The cellular levels of these proteins were unchanged (Table 3). These data indicate that dynamin regulates the secretion of a number of constitutively secreted proteins with specificity and variation between proteins.

**Table 3:** Effect of MiTMAB on macrophage protein secretion.

Protein	Secreted levels (% change)	Cellular levels (% change)
fibronectin	−87±3*	8±12
MMP-9	−65±18*	28±43
Ch3L1	−73±8*	3±9
lysozyme	−62±7*	4±8
CHC	54±32	1±15
HSP90	39±25	4±0
CypA	+191±69*	7±2

HMDM were exposed to ±30 µM MiTMAB for 2 hours.

% change in secreted and cellular protein levels were determined by Western Blotting. Data are mean ± SEM of 3–5 independent cell donors.

*P<0.05 treated *v* untreated.

Recently, studies using triple knock out mouse fibroblasts (TKO) in which dynamin I, II and III can be conditionally deleted, revealed a dynamin-independent effect of pharmacological dynamin inhibitors [34]. To ascertain whether the effects of dynamin inhibition on protein secretion were specific to the inhibition of dynamin, the effect of MitMAB, Dyngo-4a and Dynole-34-2 was examined in wild type mouse fibroblast (“Ctrl”) and in mouse fibroblasts which were dynamin-deficient (dynamin I, II, II KO; “TKO”). As apoE is not endogenously expressed, we measured the effect of dynamin status on total constitutive protein secretion using [^35^S]-pulse chase labeling of cell proteins.

Cells were treated with (TKO) and without (Ctrl) 4-hydroxytamoxifen for 7 days to induce deletion of dynamins and total [^35^S]-TCA-precipitable material in the medium was determined after treatment with 30 µM MitMAB and 30 µM Dyngo4a. Preliminary experiments identified that Dynole-34-2 appeared toxic to these cells at the concentration of 30 µM used in HMDM and CHO-apoE cells. Viability assays indicated that 10 µM was the highest non-toxic dose of this compound for these mouse fibroblasts and was used thereafter (data not shown). MitMAB and Dynole-34-2 decreased total TCA-precipitable material in the medium of Ctrl cells but not in the medium of TKO cells (Fig. 8C), whereas no effect was observed with Dyngo-4a. It has previously been described that Dyngo-4a binds to serum albumin which inhibits its efficiency of dynamin inhibition [13]. Western Blotting confirmed efficient knockout of dynamin II in TKO cells (Fig. 8B). Phosphorimager analysis of [^35^S]-secreted proteins showed decreased secretion of several proteins (Fig. 8D; indicated by the arrows) while other secreted proteins remained unchanged (Fig. 8D; indicated by asterisks) confirming that dynamin(s) regulate the secretion of specific secretory proteins. Taken together, these data indicate that, at least in part, the inhibition of constitutive protein secretion by pharmacological dynamin inhibitors requires the presence of dynamin and is not explained by off target effects.

## Discussion

This study shows for the first time that pharmacological inhibition of dynamin decreases secretion of apoE and several other constitutively secreted proteins from primary human macrophages. Using apoE as a prototype for constitutive secretion studies, we showed that its inhibition by several classes of dynamin inhibitors was dose- and time- dependent, not due to off target effects, and occurs post translationally after processing of apoE in the Golgi. Pulse chase experiments indicated that inhibition of secretion was associated with increased degradation of apoE and only a small proportion of unsecreted apoE accumulated in the cell. Live cell microscopy showed that the inhibition was associated with reduced post-Golgi movement of apoE-GFP-containing vesicles. The effect was not restricted to macrophages, was apparently not explained by changes to microtubule morphology, and was not restricted to the secretion of apoE.

Although the pharmacological inhibitors used in this study inhibit both dynamin I and dynamin II, a role for dynamin II in constitutive protein secretion is more likely. Secretion of apoE was inhibited in CHO cells which do not express dynamin I [35], no dynamin I was detected in HMDM by Western blotting (Fig. S1). In these experiments, two separate siRNA oligonucleotides directed against dynamin II inhibited the secretion of apoE from HMDM (Fig. 2). There are four dynamin II splice variants that show different cellular distribution and it has been suggested that specific functions for these splice variants exist [1], [5]. In a careful study performed in dynamin II knock out cells reconstituted with near-physiological levels of the different dynamin II variants, it was shown that although all variants could equally restore impaired clathrin-mediated endocytosis, variants ba and bb were more effective at restoring p75 exocytosis [5]. Our siRNA oligonucleotides targeted all dynamin II variants and we could therefore not determine whether protein secretion in human macrophages involves a specific or multiple specific variants. It is however, important to consider that the use of pharmacological inhibitors will affect all splice variants.

Our studies using TKO cells show that dynamin are essential for correct intracellular protein localization and secretion. Deficiency of dynamins decreased constitutive secretion of several proteins whilst the secretion of others was not affected, confirming involvement of dynamins in constitutive protein secretion.

The secretion of apoE involves microtubules and microtubule disruption decreases the secretion of apoE [29]. Dynamin II is an *in* *vitro* microtubule binding protein and regulates microtubule stability [32], [33]. As reported by others, we found that dynamin inhibition did not affect overall tubulin morphology [12]. Only MiTMAB and Dynole-34-2 consistently increased levels of acetylated tubulin, whereas they were not affected after incubation with OcTMAB, Dyngo-4a or dynasore under conditions where apoE protein secretion was markedly inhibited. This suggests either that the effect of pharmacological inhibitors of dynamin on protein secretion is not mediated via microtubule stabilization, or that overall tubulin acetylation is relatively insensitive to the stabilizing effects of dynamin inhibitors. Discrepancies between tubulin acetylation and microtubule stabilization have been previously reported [36].

MiTMAB differentially affects the secretion of various protein cargoes, inhibiting the secretion of fibronectin, MMP9, CHI3L1 and lysozyme, increasing the secretion of CypA and having no effect on secretion of CHC and HSP90. Similarly, dynamin TKO fibroblast cells demonstrated a qualitatively altered profile of constitutively secreted [^35^S]-labeled proteins indicating some selectivity in the effects of dynamin. The observation that dynamin inhibition concurrently reduces macrophage secretion of apoE, fibronectin, MMP9, CHI3L1, and lysozyme may have implications for other physiological and pathological processes, and may suggest a common vesicular carrier for these cargoes. All these proteins have been linked to inflammatory conditions, especially atherosclerosis and may affect lesion development as well as lesion stability. Fibronectin is an important component of the extracellular matrix (ECM) and mediates a wide variety of cellular interactions [37]. Plasma fibronectin but not macrophage-secreted fibronectin promotes atherosclerotic lesion development and fibrous cap formation [38]. MMP9 levels have been linked to various inflammatory conditions. As an important enzyme degrading ECM components, MMP9 plays a role in tissue remodeling, wound healing, cell migration and plaque stability [39]. As fibronectin has been shown to induce MMP9S ecretion in tumor cells [40] it is possible that some of reduced MMP9S ecretion seen in response to dynamin inhibition is secondary to its effects on fibronectin. Also associated with a wide variety of inflammatory diseases is CH3L1 [41]. CH3L1 is an inactive member of the chitinase family and has been suggested to play a role in tissue remodeling, angiogenesis and is highly induced in macrophages in atherosclerotic lesions [42]. Lysozyme release from macrophages is best known for its role in host defence because of its capacity to hydrolyse bacterial wall components [43]. In addition, lysozyme can directly interact with advanced glycation end-products, thereby preventing oxidative stress [44]. If dynamin regulates the secretion of these diverse proteins via interaction with a common vesicular carrier, its identification may allow modulation of a range of pathogenic processes simultaneously.

It is particularly interesting that dynamin inhibition increased the secretion of CypA, implying that dynamin has an important regulatory role in restricting CypA secretion. CypA belongs to a large family of intracellular peptidyl-prolyl isomerases that are involved in protein folding [45] and is secreted from various cells such as vascular smooth muscle cells, macrophages and platelets. Secreted CypA can bind to CD147 expressed on the cell surface in a autocrine manner [46] and interact with more remote targets to act as a proinflammatory cytokine that induces cell migration, proliferation, adhesion and chemotaxis and promotes atherosclerosis [47], [48]. It is possible that the increase in secreted CypA could be due to inhibition of dynamin-dependent endocytosis of CypA which has bound to its receptor CD147. Future studies will be needed to directly address this question.

In conclusion, we have demonstrated that dynamin II is involved in the constitutive secretion of apoE and other proteins released from primary human macrophages. Pharmacological manipulation of dynamin function may therefore exert complex effects on macrophage and tissue biology.

## Supporting Information

Figure S1 **HMDM and CHO-apoE cells do not express dynamin I.** 15 µg of HMDM and CHO-apoE cell lysates were separated by SDS-PAGE and dynamin I levels were detected by Western Blotting. 5 µg of a mouse brain lysate was used as a positive control.
(TIFF)

Figure S2 **Dynamin inhibition affects secretion of other constitutive secreted proteins.** HMDM were treated with 30 µM MiTMAB for 2 h. Secretion of specified proteins was determined by Western Blotting. Representative blots from triplicate cultures are shown. Samples were corrected for protein amounts of respective culture well. Quantified changes in secreted and cellular protein levels from 3–5 independent cell donors are depicted in Table 3.
(TIFF)

Movie S1 **Movement of apoE-containing vesicles in control cell.** HMDM were transiently transfected with apoE-GFP and cultured for 24 h prior to performing live cell microscopy. Individual cells expressing apoE-GFP were identified and tracked for 3–5 min. HMDM were then treated with 30 µM MiTMAB or Dynole-34-2 for 15–30 min prior to imaging.
(AVI)

Movie S2 **Movement of apoE-containing vesicles after MitMAB exposure.** Same cell identified for Movie S1 was exposed to 30 µM MiTMAB and imaged after 15–30 m min.
(AVI)

Movie S3 **Movement of apoE-containing vesicles in control cell.** HMDM were transiently transfected with apoE-GFP and cultured for 24 h prior to performing live cell microscopy. Individual cells expressing apoE-GFP were identified and tracked for 3–5 min. HMDM were then treated with 30 µM MiTMAB or Dynole-34-2 for 15–30 min prior to imaging.
(AVI)

Movie S4 **Movement of apoE-containing vesicles after Dynole34-2 exposure.** Same cell identified for Movie S3 was exposed to 30 µM Dynole34-2 and imaged after 15–30 m min.
(AVI)
