# Supplementary figures and images for: Pharmacological Inhibition of Dynamin II Reduces Constitutive Protein Secretion from Primary Human Macrophages
Source: PLoS One. 2014 Oct 27;9(10):e111186. doi: 10.1371/journal.pone.0111186 (PMC4210248; doi:10.1371/journal.pone.0111186)

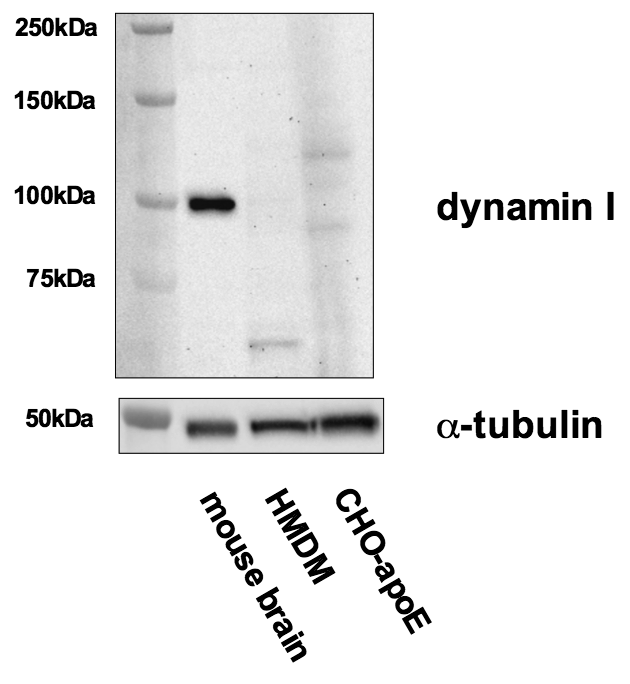

Supplement: Figure S1 — HMDM and CHO-apoE cells do not express dynamin I. 15 µg of HMDM and CHO-apoE cell lysates were separated by SDS-PAGE and dynamin I levels were detected by Western Blotting. 5 µg of a mouse brain lysate was used as a positive control. (TIFF) [file pone.0111186.s001.tiff]

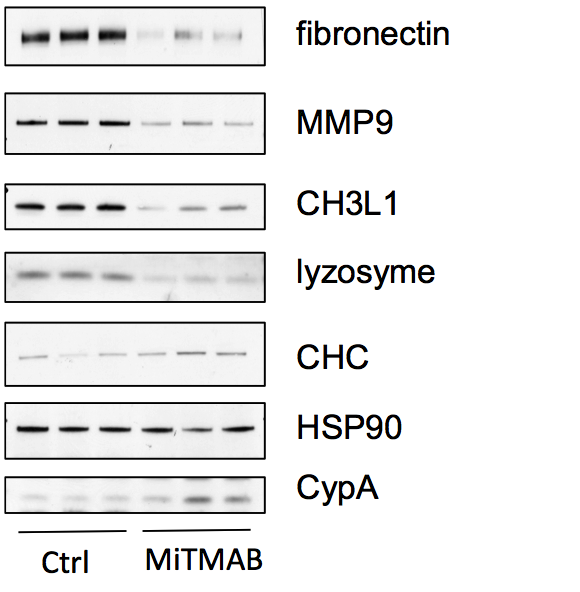

Supplement: Figure S2 — Dynamin inhibition affects secretion of other constitutive secreted proteins. HMDM were treated with 30 µM MiTMAB for 2 h. Secretion of specified proteins was determined by Western Blotting. Representative blots from triplicate cultures are shown. Samples were corrected for protein amounts of respective culture well. Quantified changes in secreted and cellular protein levels from 3–5 independent cell donors are depicted in Table 3. (TIFF) [file pone.0111186.s002.tiff]
